# *In vitro* shoot regeneration from crown and sucker explants of *Ananas comosus* cv. Josapine

**DOI:** 10.21315/tlsr2025.36.3.7

**Published:** 2025-10-31

**Authors:** Dahmendra Sriskanda, Soo Ping Khor, Li Vern Tan, Sreeramanan Subramaniam, Nurulhikma Md Isa, Bee Lynn Chew

**Affiliations:** 1 School of Biological Sciences, Universiti Sains Malaysia, 11800 USM Pulau Pinang, Malaysia; 2 Department of Biological Sciences and Biotechnology, Faculty Science and Technology, Universiti Kebangsaan Malaysia, 43600 UKM Bangi, Selangor, Malaysia

**Keywords:** *Ananas comosus*, Josapine, BAP, Kinetin, Pineapple, Ananas comosus, Josapine, BAP, Kinetin, Nanas

## Abstract

Pineapple, *Ananas comosus* (L.) Merr., is a highly nutritious, major tropical Amazonian fruit valued for its health properties. Josapine, a successfully commercialised Malaysian hybrid, bears sweet fruits and has a shorter gestation period as compared to other cultivars. The current study aims to evaluate the in vitro regeneration and shoot multiplication potential of Ananas comosus cv. Josapine using different types of explant sources and different concentrations of cytokinins. Pineapple crown and suckers were surface sterilised and cultured in MS medium supplemented with 6-Benzylaminopurine (BAP) at different concentrations (0.0 mg/L, 1.0 mg/L, 2.0 mg/L and 3.0 mg/L) for six weeks to evaluate the shoot regeneration efficiency of both explants used. In vitro induced shoots from crown and sucker explants were also cultured in different concentrations of BAP and kinetin (0.0 mg/L, 0.5 mg/L, 1.0 mg/L, 1.5 mg/L, 2.0 mg/L, 2.5 mg/L and 3.0 mg/L) for the induction of multiple shoots and roots. Results from the current study revealed that the highest percentage of shoot induction obtained from crown explants was 88.00% in the treatment of 2.0 mg/L BAP (3.50 ± 0.67 shoots per explant), whereas the treatment of 3.0 mg/L BAP resulted in the highest percentage of shoot induction (82.00%) from sucker explants. As for the response of in vitro shoots, the treatment of 2.0 mg/L BAP resulted in the highest number of shoots (6.85 ± 0.61 shoots per explant), whereas the highest number of roots (6.09 ± 0.46 roots per explant) was observed in MS media supplemented with 1.5 mg/L kinetin after 16 weeks of culture. This study demonstrates methods for in vitro shoot multiplication suitable for the micropropagation and commercialisation of the Josapine cultivar. This facilitates the mass production of disease-free, high-quality planting materials, leading to improved fruit quality and enhanced export potential of this pineapple cultivar.

## Abstract (fr)

**Abstrak:**

Nanas, Ananas comosus (L.) Merr., ialah buah yang berasal dari Amazon tropika utama yang berkhasiat dan bernilai. Josapine, hibrid Malaysia yang berjaya dikomersialkan, menghasilkan buah yang manis dan mempunyai tempoh kematangan yang lebih pendek berbanding kultivar lain. Kajian semasa bertujuan untuk menilai potensi regenerasi in vitro dan potensi penggandanan pucuk Ananas comosus cv. Josapine menggunakan sumber eksplan dan kepekatan sitokinin yang berbeza. Jambul dan sulur nanas diisteril dan dikultur dalam medium MS dengan 6-Benzylaminopurine (BAP) pada kepekatan berbeza (0.0 mg/L, 1.0 mg/L, 2.0 mg/L dan 3.0 mg/L) selama enam minggu untuk menilai kecekapan penjanaan semula pucuk dari dua jenis eksplan yang digunakan. Pucuk yang tumbuh daripada eksplan jambul dan sulur juga dikultur dalam kepekatan BAP dan kinetin yang berbeza (0.0 mg/L, 0.5 mg/L, 1.0 mg/L, 1.5 mg/L, 2.0 mg/L, 2.5 mg/L dan 3.0 mg/L) untuk penginduksian pucuk dan akar. Hasil kajian mendedahkan bahawa peratusan tertinggi aruhan pucuk yang diperoleh daripada eksplan jambul ialah 88.00% dalam rawatan 2.0 mg/L BAP (3.50 ± 0.67 pucuk setiap eksplan), manakala rawatan 3.0 mg/L BAP menghasilkan peratusan tertinggi penginduksian pucuk (82.00%) daripada eksplan sulur. Bagi tindak balas pucuk in vitro, rawatan 2.0 mg/L BAP menghasilkan bilangan pucuk tertinggi (6.85 ± 0.61 pucuk setiap eksplan), manakala bilangan akar tertinggi (6.09 ± 0.46 akar setiap eksplan) diperhatikan dalam media MS ditambah dengan 1.5 mg/L kinetin selepas 16 minggu kultur. Kajian ini menunjukkan kaedah pengandaan pucuk in vitro yang sesuai untuk mikroperambatan dan pengkomersilan kultivar Josapine. Ini memudahkan pengeluaran skala besar bahan tanaman bebas penyakit dan berkualiti tinggi, yang membawa kepada kualiti buah yang lebih baik dan potensi eksport tinggi bagi kultivar nanas ini.

**HIGHLIGHTS**

- Induction of multiple shoots from crown and sucker explants of *Ananas comosus* cv. Josapine.
- Crown and sucker explants of regenerated multiple shoots in treatments of BAP.
- BAP treatments induced multiple shoots whereas kinetin induced roots from in vitro shoots *Ananas comosus* cv. Josapine

## INTRODUCTION

The pineapple, scientifically known as *Ananas comosus* (L.) Merr. originates from the Amazon basin and is highly prized for its rich nutritional profile and lively tropical taste. This fruit falls within the category of major fruits and ranks as the third most extensively cultivated fruit crop, thriving across more than 82 countries, covering over 2.1 million acres for its cultivation and development (Hossain 2016). Pineapples account for more than 20% of total tropical fruit production in the world (Wijeratnam 2016). Thailand plays a massive role in global pineapple production, contributing a staggering 13% of the total output, making it the top pineapple-producing country in the world (Balogun *et al*. 2018). Malaysia produced 272,570 metric tons of pineapple throughout the year 2015, according to the Department of Agriculture (DoA) (Suhaimi & Fatah 2019). Pineapples are widely enjoyed for their versatility in consumption. They can be incorporated into Malaysian cuisine through cooking, enhancing the flavours of various dishes, used as toppings on pastries, processed into jams and juices or enjoyed fresh. The taste profile of pineapples encompasses a delightful blend of sweet, fruity, fresh, floral, green, apple-like and woody notes (Lasekan & Hussein 2018). The utilisation of young pineapple juice in treatments for various conditions, including bronchitis, gum issues and bowel diseases, is attributed to the presence of essential nutrients like bromelain, as well as vitamins A and B1 (Halim 2016). In addition, pineapple also contains citric acid that can effectively eliminate fat and help in reducing weight (Hossain *et al*. 2015; Halim 2016). Patients with nervous system complications benefit from adequate thiamine intake in lowering metabolic alterations caused by diabetes and glucose levels, as well as in increasing red blood cell formation (Cannon & Ho 2018). Bromelain, a proteolytic enzyme found in pineapple fruit, aids in digestion and is used for its therapeutic properties due to being anti-inflammatory, antioxidant, anti-cancer and cardioprotective agent (Zdrojewicz *et al*. 2018). Bromelain was also combined with analgesics in the treatment of acute thrombophlebitis, as well as in the regulation of myocardial infarction (Priyadarshani *et al*. 2019). Moreover, pineapple is recognised for its antioxidant properties, primarily due to phenolics present in its composition, including methanol (51%), ethyl acetate (14%) and water extract (3%) (Hossain & Rahman 2011). Pineapples harbour volatile compounds composed of 35% esters, 26% ketones, 18% alcohols, 9% aldehydes, 3% acids and 9% other compounds (De Oliveira Barretto *et al*. 2013).

Moris, MD2, Josapine, N36 and Sarawak are the most popular pineapple varieties in Malaysia, and they are commonly grown in Johor, Sarawak, Sabah, Kedah, Selangor, Negeri Sembilan, Pahang and Terengganu (Suhaimi & Fatah 2019). The Josapine pineapple, a commercially successful cultivar developed in Malaysia, is cultivated primarily for fresh consumption. It originated from crossbreeding the female Johor pineapple with the male Sarawak pineapple. The Josapine typically boasts an average fruit size ranging from 1.1 kg to 1.3 kg, displaying an attractive orange-red hue when ripe. It offers a sweetness level falling between 17% to 22% Brix, showcasing substantial potential for export (Shamsudin *et al*. 2007). The fruit can be harvested within 10 months, providing approximately 2–3 weeks of shelf life. Josapine is also highly tolerant towards diseases such as bacterial heart rot and fruit collapse (Shamsudin *et al*. 2007). In a study conducted on the presence of bioactive compounds and antioxidant capacity, the highest total phenolic and tannin content was observed in Josapine pineapples, followed by Morris and Sarawak varieties indicating the health potentiality of the Josapine pineapple (Chiet *et al*. 2014). Abdul Hamid and Ali (2014) reported that 92.4% local farmers were contented with its yield and market value, with an added advantage of the variety producing the sweetest pineapples and having a shorter gestation period as compared to other cultivars. The Josapine cultivar holds significant importance within the local agricultural economy, particularly in both the fresh fruit and processed fruit markets.

Traditional vegetative propagation methods for pineapples typically include excising crowns into multiple vertical pieces, collecting slips from the fruit base, and harvesting both lateral and basal shoots from the pineapple plant (Reinhardt *et al*. 2018). Nevertheless, these traditional methods do not guarantee consistent mass propagation or simultaneous harvesting as these propagules grow at different phases and flower at different times naturally (Shu *et al*. 2019). Therefore, micropropagation techniques are often employed to accelerate the production of propagules obtained individually from specific plant parts such as crowns or suckers. This method aims to reduce the occurrence of ‘natural flowering’ and synchronise the fruiting process (DeWald *et al*. 1992). Plant tissue culture is an alternative in plant propagation which constitutes of in vitro techniques, enabling mass propagation of desired plants in a media supplemented with plant growth regulators under controlled conditions. Plant propagation via micropropagation has been established for many commercial crops including various pineapple cultivars such as Maspine (Zuraida *et al*. 2011), Josapine (Awal *et al*. 2011), Moris (De Silva *et al*. 2008), Sipahutar (Insani *et al*. 2018) and Kew (Nikumbhe 2013) cultivars. This process is essential to meet the demand of growers and align with market expectations efficiently (Pamela *et al*. 2014). Sulaiman *et al*. (2020) investigated on the combined effects of plant growth regulators for the MD2 cultivar and reported that MS medium containing 30 g/L sucrose without plant growth regulators resulted in the most significant shoot regeneration approximately 2.80 ± 0.5 shoots, whereas MS medium supplemented with 30 g/L sucrose and 1.0 mg/L NAA showed the highest increase in plantlet height, reaching approximately 4.40 ± 0.3 cm. Devi *et al*. (2018) on the other hand, reported that the combination treatment of 2 mg/L BAP with 2 mg/L NAA in MS media led to the highest number of shoots (22.71) for the Kew pineapple variety. Meanwhile, for shoot elongation, the combination of 0.5 mg/L BAP and 2 mg/L NAA resulted in growth of approximately 8.09 cm after 90 days from inoculation (Devi *et al*. 2018). Besides, the utilisation of temporary immersion systems on the micropropagation of pineapples has been explored in literatures, wherein nodule cluster cultures were subjected to phytohormone treatments to induce microshoots (shoots smaller than 0.5 cm). For example, Scherer *et al*. (2013) reported the highest microshoot induction (65.0%) with the treatments of 2 μM NAA and 8 μM 2-iP or BAP, and maximum elongation of microshoots at 25.5 cm with the employment of 10 μM GA_3_. Whereas, in the micropropagation of *Ananas comosus* var. *Bracteatus* L. (ornamental pineapple), MS medium supplemented with 2 mg/L BAP in temporary immersion bioreactor produced the highest shoot number at 7.07 shoots (Reis *et al*. 2018).

As for reports on the micropropagation of Josapine, Awal *et al*. (2011) reported that optimal regeneration was achieved on MS medium supplemented with 3.0 mg/L BAP and on MS medium containing 1.0 mg/L BAP combined with 0.5 mg/L NAA. The shoots derived from crown tip meristems were further optimised using MS liquid medium (Awal *et al*. 2011). Since then, published research has been scarce on the micropropagation of the Josapine cultivar, particularly regarding the exploration of other propagation materials namely Josapine pineapple suckers as a source of explants. Considering its substantial potential within the local agriculture industry, the current study aims to evaluate in vitro multiple shoot induction of the Josapine pineapple cultivar utilising different sources of explants and cytokinins (BAP and Kinetin) for the induction of multiple shoots with the aim to facilitate the production of healthy plant stocks suitable for commercialisation through micropropagation. The development of an effective micropropagation protocol for the Josapine cultivar could facilitate the rapid production of uniform, disease-free planting materials to support the Malaysian pineapple industry whereby established protocols could be further adapted for other new cultivars such N36, Morris Gajah and Yankee.

## MATERIALS AND METHODS

### Surface Sterilisation of Explants

The crowns and suckers of field-grown pineapples were obtained from Arus Primajaya Sdn. Bhd. in Johor, Malaysia. The explants were defoliated by removing the first few outer layers prior to the surface sterilisation treatment. This process targeted the elimination of the leafy top and the fibrous basal portion of both crowns and suckers. Following this, the crowns and suckers were thoroughly washed under tap water to eliminate any debris, followed by a rinse with distilled water. The defoliated crowns and suckers, measuring 4 cm–5 cm in height, were then brought into a sterile environment in preparation for surface sterilisation. The crown and sucker explants were submerged in 70% Ethanol for 2 min, followed by two-step Clorox© treatment (40% Clorox© solution) for 10 min–12 min. This is then followed by 20% Clorox© solution for 7 min, supplemented with a few drops of Tween 20 solution. The explants were then rinsed with autoclaved double distilled water 3 times and the sterilised explants were further defoliated to reveal the primordial leaves and necrotic segments of the explants were removed.

### Shoot Induction from Crown and Sucker Explants

Surface sterilised crown explants were inoculated into solid Murashige and Skoog (MS) medium (Murashige & Skoog 1962) supplemented with different concentrations of BAP at 0.0 mg/L, 1.0 mg/L, 2.0 mg/L and 3.0 mg/L. Whereas, sucker explants were cultured into MS medium without the supplementation of BAP for 4 weeks prior to inoculation into MS medium incorporated with different concentrations of BAP (0.0 mg/L, 1.0 mg/L, 2.0 mg/L and 3.0 mg/L). Each treatment has six explant replicates, and the experiment was repeated twice. The data of the number of induced shoots was collected at week six of culture. The evaluated parameters were the percentage of shoot induction towards different concentrations of phytohormone, and the average number of shoots induced per explant.

### Shoot and Root Induction from *In Vitro* Shoot Explants

*In vitro* induced shoots of eight weeks old were excised and inoculated into solid MS media added with different concentrations of BAP and Kinetin (0.0, 0.5, 1.0, 1.5, 2.0, 2.5 and 3.0 mg/L). Each treatment has eight explant replicates, and the experiment was repeated twice. All explants were sub-cultured at week 8 into the same treatment media for further observation. The formation of shoots and roots of the crown and sucker explants were monitored every two weeks and parameters such as the percentage of explant regeneration towards phytohormones and the number of induced shoots and roots were collected at week 16 of culture.

### Growing Conditions

All explants were maintained in the culture room with controlled environment of 16:8 hour light:dark cycle of photoperiod under white LEDs (light-emitting diodes) light (Philips TLD 36W/865 – 6500K, 3070 lm), with the temperature of 25 ± 2°C and humidity level at 50 ± 10% (Extech, USA).

### Data Analysis

Data analysis was performed by using Statistical Package for the Social Sciences (SPSS) version 26. All data obtained were subjected to one-way variance (ANOVA), followed by Duncan’s Multiple Range Test at the significance of *p* < 0.05.

## RESULTS AND DISCUSSION

### Shoot Induction from Crown and Sucker Explants

In this study, the treatment of MS medium supplemented with 2.0 mg/L BAP resulted in the highest percentage of shoot induction and the highest number of shoots induced for the crown explants with the value of 88.00% and 3.50 ± 0.67 shoots per explant, respectively (Table 1). However, the value for the average number of shoots was not significant compared to the other treatments except for the control (*p* < 0.05) whereby the control treatments were unsuccessful in generating in vitro shoots. This observation was attributed to severe explant browning, ultimately resulting in the mortality of the crown explants. As for the sucker explants, the highest percentage of shoot induction (82.00%) was observed in the treatment of 3.0 mg/L BAP. Nevertheless, the supplementation of 1.0 mg/L and 2.0 mg/L BAP resulted in the highest number of induced shoots with the values of 2.50 ± 0.62 and 2.50 ± 0.43 shoots per explant respectively. These results were also not significant compared to the control and 3.0 mg/L BAP. The lowest value of shoots was observed in the control treatment with only 1.50 ± 0.34 shoots/explant induced from the sucker explants.

**FIGURE 1:**
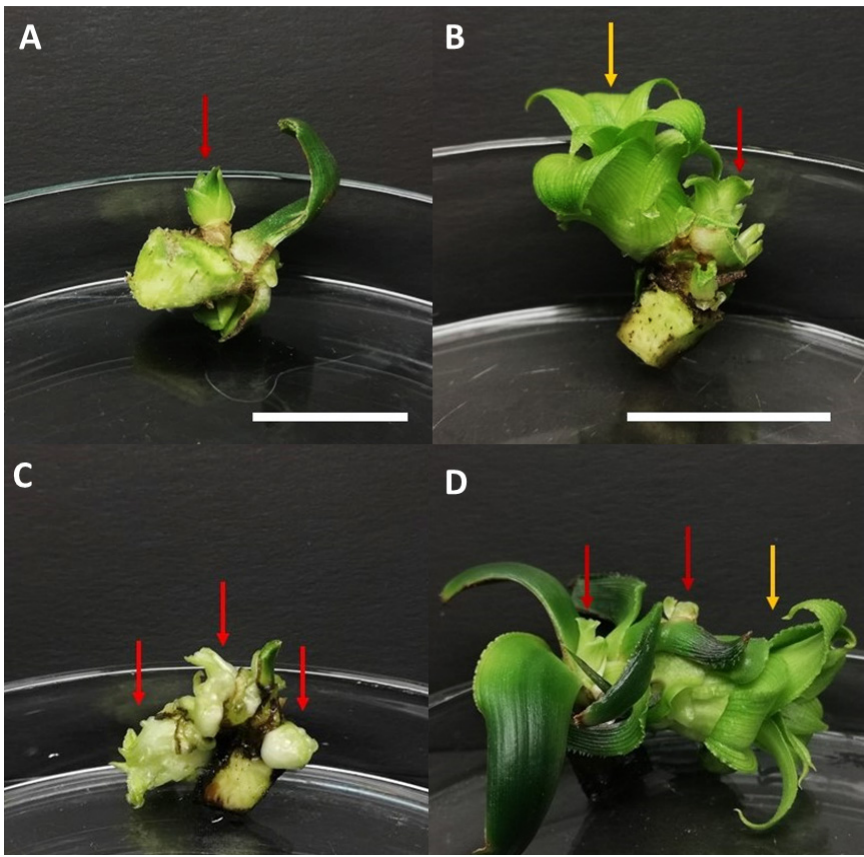
Surface sterilised sucker explants in BAP treatments after six weeks of culture. (A) Control; (B) MS medium supplemented with 1.0 mg/L BAP; (C) MS medium with 2.0 mg/L BAP; and (D) MS medium with 3.0 mg/L BAP. Newly formed shoots were evident for the treatments of 1.0 and 2.0 mg/L BAP. Red arrows indicate the formation of micro shoots, whereas the yellow arrows indicate well-developed macro shoots. Bars = 2 cm.

The findings of the current study are in synchrony with previous literature, whereby multiple shoots in Ananas comosus can be induced by the application of BAP solely without a combination of other plant growth regulators (Badou *et al*. 2018). The highest number of shoots (3.50 ± 0.67) was obtained in the incorporation of 2.0 mg/L BAP into MS medium when surface sterilised explants were used, whereas Nikumbhe (2013) reported 3.20 ± 0.31 shoots when 1.5 mg/L BAP and 0.5 mg/L NAA were used in the Kew cultivar. Pineapple crowns originate from the apical meristem of the pineapple fruit, while suckers originate from the axillary buds located at the base of the plant (Reinhardt *et al*. 2018). In plants, the apical meristem synthesises endogenous auxin to inhibit lateral bud growth (Bhatia *et al*. 2015). Nonetheless, the addition of cytokinin at high levels in vitro synergistically reacts with the auxin naturally present at the apical meristem. Therefore, apical dominance is suppressed which facilitates more explants to react to the synergy, thereby inducing multiple shoots. Zhang *et al*. (2023) noted that crown explants of shredded pineapple were more suitable compared to suckers for shoot induction with MS media supplemented with 3.0 mg/L BAP and 3.0 mg/L NAA being the best medium for shoot induction and MS media with 3.0 mg/L BAP and 1.0 mg/L NAA for shoot proliferation. In their study on Smooth Cayenne pineapple crowns, Usman *et al*. (2013) reported the production of 11.5 and 14.4 plantlets when cultured on MS medium supplemented with 5 μM BAP and 3 μM NAA, respectively. In contrast, sucker explants were able to produce *in vitro* shoots without the supplementation of BAP (Fig. 1), possibly due to the higher levels of endogenous cytokinin present in pineapple suckers. In this study however, the supplementation of BAP was necessary to induce shoots from sucker explants which was also reported in other findings. Be and Debergh (2006) induced 8 to 9 shoots from sucker explants in liquid MS medium added with 0.8 mg/L–1.0 mg/L BAP upon 8 weeks of culture. On the other hand, Ab Rahman *et al*. (2021) included organic additives such as coconut water to boost the regeneration of shoots from sucker explants for the variety of MD2. Their results revealed that the culture medium supplemented with 2.0 mg/L BAP, 1.0 mg/L NAA and 20.00 mL/L coconut water produced the highest number of shoots, with an average of 45.33 ± 7.56. Alternatively, a study by Rosmaina *et al*. (2024) reported that the application of 7.5 mL/L AB Mix as an alternative to MS media, with the supplementation of 2 ppm BAP and 0.5 ppm NAA resulted in the fastest shoot emergence (30.55 days) and the highest number of shoots (3.55 shoots per explant) from bud explants of the Queen cultivar.

**FIGURE 2:**
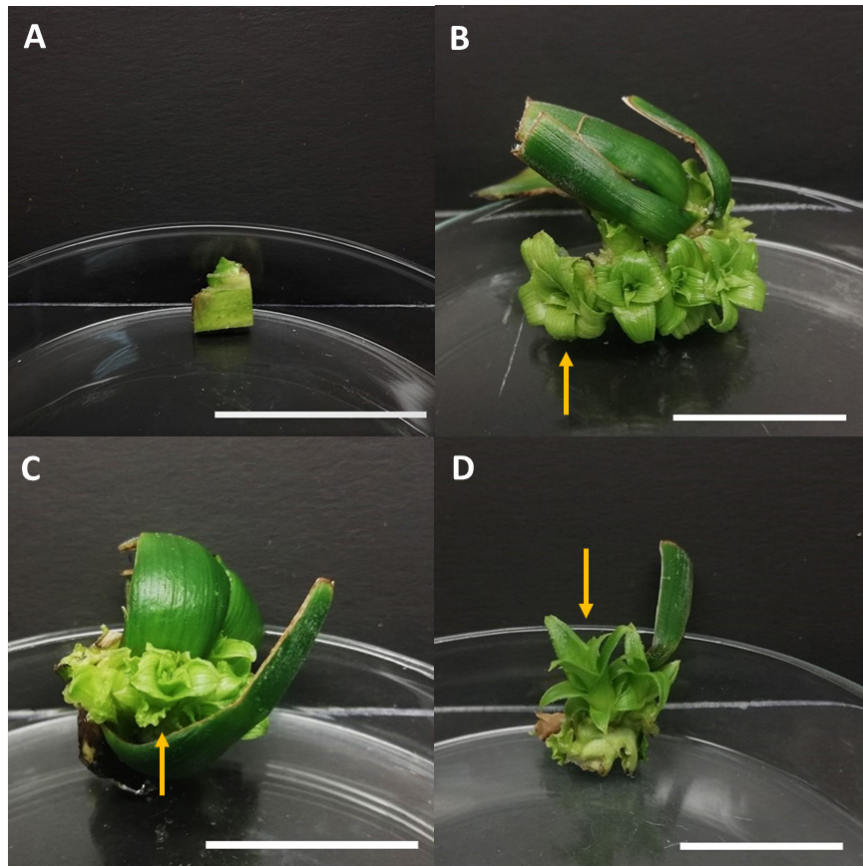
Surface sterilised crown explants in BAP treatments after six weeks of culture. (A) Control; (B) MS medium added with 1.0 mg/L BAP; (C) MS medium added with 2.0 mg/L BAP; and (D) MS medium added with 3.0 mg/L BAP. Newly formed shoots were also evident for the treatments of 1.0 and 2.0 mg/L BAP. Yellow arrows indicate the formation of well-developed macro shoots. Note that the Scale bars = 2 cm.

Culture medium browning is another issue encountered in this study. The pineapple crown is highly susceptible to senescence as it is structurally the shoot apex of the fruit, therefore, a higher phenolic compound content (Azizan *et al*. 2020) accounts for the observable medium browning and growth retardation. This has also possibly been attributed to the inability of the crown explants (particularly the crown leaves) to adapt to the *in vitro* conditions as observed in control (Fig. 2a). Andrade *et al*. (2000) and Sedighi *et al*. (2014) mentioned that smaller-sized crown explants are highly susceptible to browning especially when they are excised, thereby causing lesions that increase the release of polyphenols which would alter the culture medium composition and interfere with nutrient absorption (Souza *et al*. 2018). According to Sato *et al*. (2001) and Ozyigit *et al*. (2007), explant growth is inhibited when toxic substances are released by the oxidisation of phenolic compounds by polyphenol enzymes (Souza *et al*. 2018). Their findings were in agreement with this study, wherein browning due to the phenolic compounds released by crown and sucker explants occurred in the culture medium. Therefore, it was established that the inclusion of BAP was beneficial in aiding surface-sterilised crown explants to induce multiple shoots. Conversely, MS medium without BAP supplementation proved adequate for the production of *in vitro* shoots in sucker explants.

### Regeneration of Shoots and Roots from *In Vitro* Shoot Explants

#### Regeneration of shoots and roots with BAP supplementation

The results obtained indicated no statistical significance for shoot induction of in vitro shoot explants for all treatments of BAP. Based on Table 2, 2.5 mg/L BAP resulted in the highest percentage of shoot induction from the *in vitro* shoot explants (87.50%). The total number of 6.85 ± 0.61 shoots/explant was observed in the treatment of MS medium added with 2.0 mg/L BAP, which was significantly different (*p* < 0.05) compared to the control (1.57 ± 0.43 shoots/explant). The highest number of roots was induced in the control treatment (4.82 ± 0.39 roots/explant), whereas, 2.0 mg/L and 3.0 mg/L BAP resulted in no root formation.

**FIGURE 3:**
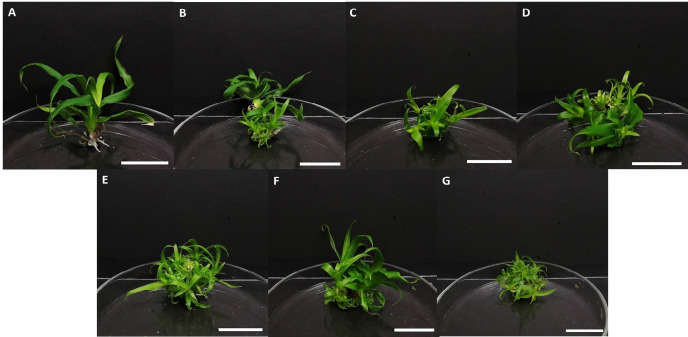
The induction of shoots from *in vitro* shoot explants in different treatments of BAP after 16 weeks of culture. MS media supplemented with BAP at (A) 0.0 mg/L; (B) 0.5 mg/L; (C) 1.0 mg/L; (D) 1.5 mg/L; (E) 2.0 mg/L; (F) 2.5 mg/L; and (G) 3.0 mg/L. Scale bars = 2 cm.

Organogenesis was observed to induce at a slower rate when in vitro shoots were used as explants, as opposed to the crown and sucker explants obtained from ex vitro sources. This is because *in vitro* plantlets over a series of subculture would be less receptive towards the production of endogenous phytohormones as opposed to surface sterilised explants, as exogenous hormones would interfere with the ability of the cells to synthesise, accumulate and distribute endogenous hormones for induction and proliferation (Su & Zhang 2014; Cai *et al*. 2018). This rationale justifies the subculture at week eight of treatment into the same media to promptly initiate the production of multiple shoots. The *in vitro* explants demonstrated a quiescent period with no morphological responses during the initial eight weeks of culture, regardless of the treatments applied. Additionally, the proliferated shoots did not exhibit shoot tip necrosis in the first eight weeks of culture on the same medium, which is in agreement with Lakho *et al*. (2023). Various studies have reported on the *in vitro* shoot production in *Ananas comosus* with the application of different plant growth regulators. Shoot regeneration was observed in a study on Maspine pineapple whereby MS medium with 5.0 mg/L BAP resulted in 86% shoot regeneration (Zuraida *et al*. 2011). Khan *et al*. (2004) reported a total of 3.85 ± 0.58 shoots per bud explant of Ananas comosus using a combination of 0.5 mg/L BAP and 0.001 mg/L NAA. Awal *et al*. (2011) on the other hand, studied different combinations of plant growth regulators and concentrations to regenerate shoots by using the crown tip meristems of the Josapine cultivar as explants. In their study, the highest number of shoots (3.00 ± 0.63) was observed in the treatment of MS medium supplemented with 3.0 mg/L BAP, whereas combination treatments of 1.0 mg/L BAP and 0.5 mg/L NAA resulted in the induction of 4.00 ± 0.35 shoots. Nevertheless, the current study observed the highest production of the average number of shoots (6.85 ± 0.61 shoots) in the treatment of 2.0 mg/L BAP without the incorporation of auxins (Fig. 3), which were higher than the findings of previous studies mentioned above.

Rooting In *in vitro* regeneration, BAP is commonly used due to its effectiveness in promoting organogenesis and somatic embryogenesis (Carimi & De Pasquale 2003). According to Cárdenas-Aquino *et al*. (2023), BAP enhances shoot proliferation by upregulating genes associated with cell division, stem cell maintenance in the shoot apical meristem, and cytokinin signalling pathways, contributing to improved shoot development. Its common use is further supported by its availability, cost-effectiveness, and strong biological activity. BAP is commonly applied for shoot induction and in the micropropagation protocols of different pineapple cultivars. Shoot induction was observed to be the highest (5.00 ± 0.20) when MS medium with 3.0 mg/L BAP was employed, in a study conducted on sucker explants of the MD2 pineapple (Hamid *et al*. 2013). In the *in vitro* shoot induction of ‘Yeppoon Gold’ which is a clone of Smooth Cayenne after four weeks of inoculation, MS medium added with 1.0 mg/L BAP rapidly initiated the formation of shoots and the highest proliferation when inoculated in B5 medium at 2.0 mg/L BAP (Bhatia & Ashwath 2002). An average shoot number of 6.1 per explant in the Smooth Cayenne variety was observed when semi-liquid MS basal medium was enriched with 1.5 mg/L BAP, for five weeks (Pamela *et al*. 2014). Atawia *et al*. (2016) obtained the highest shoot multiplication (56 shoots per explant at first subculture and 42 shoots per explant at the second subculture) when the full-strength MS media was equipped with 2.0 mg/L BAP, using the Smooth Cayenne variety. Furthermore, a recent study that involved culturing buds from *in vitro* Sipahutar pineapples in MS medium supplemented with 1.0 mg/L IAA and 4.0 mg/L BAP resulted in the production of only 1.43 shoots (Insani *et al*. 2018). Based on these reports, the current study indicated that BAP alone at 2.0 mg/L BAP is adequate to stimulate the induction of shoots from the in vitro shoots of Josapine cultivar as part of the multiplication step in micropropagation.

#### Regeneration of shoots and roots with Kinetin supplementation

**FIGURE 4:**
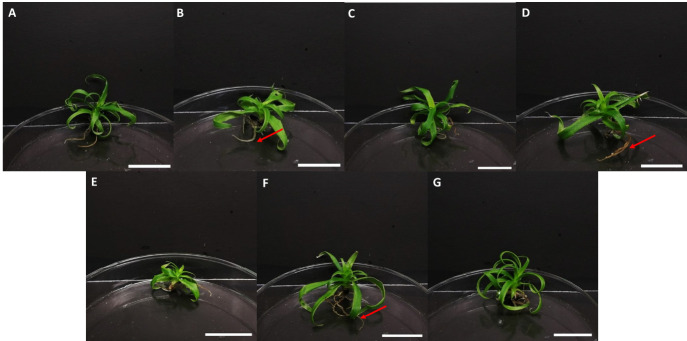
The induction of shoots from *in vitro* shoot explants in different treatments of BAP after 16 weeks of culture. MS media supplemented with BAP at (A) 0.0 mg/L; (B) 0.5 mg/L; (C) 1.0 mg/L; (D) 1.5 mg/L; (E) 2.0 mg/L; (F) 2.5 mg/L; and (G) 3.0 mg/L. Scale bars = 2 cm.

Shoot induction was proven to be less efficient when the in vitro shoots were supplemented with various concentrations of kinetin, in this study. In terms of explant growth, kinetin supplementation resulted in maturation and elongation of explant alongside increased root induction (Fig. 4). The highest percentage of explant regeneration (90.63%) was observed in the control treatment, whereas maximum shoot induction (3.50 ± 0.96 shoots per explant) was observed in the treatment of 2.5 mg/L kinetin. In terms of root induction, the inclusion of kinetin at a concentration of 1.5 mg/L resulted in the highest number of roots (6.09 ± 0.46). However, while this value was not significantly different from other kinetin treatments tested, it was notably significant (*p* < 0.05) compared to the control in terms of root production (Table 3).

Rooting of *in vitro* explants is a crucial aspect of micropropagation as it positively influences the acclimatisation and survival of the explants beyond laboratory conditions (Shukla *et al*. 2020). Previous studies have reported on the supplementation of various auxins in MS medium for in vitro rooting. In a study conducted on the Smooth Cayenne variety, MS medium supplemented with 1.0 mg/L IAA resulted in the highest number of roots at 10.67 roots per shoot explant (Atawia *et al*. 2016). Khan *et al*. (2004) reported that the average number of roots at 5.00 was observed in treatments of 1.0 mg/L IBA in their pineapple micropropagation study. In a previous study, MS medium supplemented with 1.0 mg/L NAA produced the highest number of roots at 9.23 after one month of inoculation in the study on crown meristems and slips of the Tainong 11, Tainong 21 and MD2 pineapple varieties (Priyadarshani *et al*. 2018).

However, in terms of shoot induction, the findings of this study is in contrast with the findings of Ibrahim *et al*. (2013) whereby explants of the Queen cultivar inoculated in MS medium supplemented with 1.0 mg/L Kinetin resulted in 18.60 shoots per explant. The possibility of such disparities can be attributed to the fact that different plant cultivars have varying genotypes that respond differently when subjected to exogenous phytohormones (Bhushan *et al*. 2017). The results of the current study contradict the established notion that kinetin typically exerts an inhibitory effect on *in vitro* explants. A relatively high auxin to kinetin ratio promotes rooting, whereas a higher kinetin to auxin ratio favours shoot formation (Smith 2013). Conventionally, kinetin is known to counteract the action of auxin, leading to growth inhibition and the cessation of root formation (Yaacob & Mat Taha 2017). However, the findings from this study diverge from the anticipated effect of kinetin on root formation in in vitro explants. This divergence may be attributed to the receptivity of pineapple explants to kinetin, potentially triggering gene expression that promotes root formation. While kinetin may not stimulate shoot production as effectively as BAP for the Josapine cultures, it was found to indirectly stimulated the formation of roots. This study has evaluated the response of different explant source and the induction of multiple shoots using BAP and Kinetin for the establishment of micropropagation methods on the Josapine pineapple cultivar. While pineapple propagation via plant tissue culture could be an efficient alternative in the production of high-quality plants for farms, its effectiveness is influenced by genotype, physiological variation, and external conditions. Some pineapple genotypes may have poor regeneration capacity, slower growth rates, or may be more sensitive to media compositions and hormonal treatments, indirectly influencing the applicability of standardised protocols across all cultivars. Hence justifying the importance of an optimised protocol for the various cultivars available.

## CONCLUSION

From this study, optimal production of *in vitro* shoots from *ex vitro* explants of the Josapine cultivar was achievable through the employment of 2.0 mg/L BAP for crown (3.50 ± 0.67 shoots) and sucker (2.50 ± 0.43 shoots) explants, thereby rendering the utilisation of two alternative pineapple propagative materials to conduct micropropagation of this cultivar. In terms of shoot induction from in vitro shoot explants, the treatment with 2.5 mg/L BAP and Kinetin resulted in the highest percentage of explant regeneration, both with a value of 87.50%. After 16 weeks of culture, MS medium supplemented with 2.0 mg/L BAP produced the highest number of shoots (6.85 ± 0.61), whereas MS media supplied with 1.5 mg/L kinetin produced the highest number of roots (6.09 ± 0.46). The methods reported in this study contribute to the ongoing research on this cultivar and, indirectly, enhance the production and steady supply of uniform and disease-free planting materials of high-quality, disease-free pineapple plant stocks for the agricultural industry. Consequently, it plays a crucial role in meeting the demands of the Malaysian agricultural sector by enhancing productivity, promoting sustainable cultivation practices, and reducing dependence on conventional propagation methods that are often slower and more susceptible to pests and diseases.

## Figures and Tables

**TABLE 1:** Percentage of shoot induction and the average number of shoots of pineapple crowns and suckers after six weeks of inoculation in MS medium supplemented with BAP.

Concentration of BAP (mg/L)	Percentage of shoot induction (%)	Average no. of shoots, N ( *x* ± s.e)
Crown explants		
0.0	0.00	0.00 ± 0.00^b^
1.0	50.00	2.00 ± 1.00^a^
2.0	88.00	3.50 ± 0.67^a^
3.0	83.00	2.60 ± 0.68^a^
Sucker explants		
0.0	75.00	1.50 ± 0.34^a^
1.0	60.00	2.50 ± 0.62^a^
2.0	64.00	2.50 ± 0.43^a^
3.0	82.00	2.13 ± 0.44^a^

*Notes:*

* Data followed by different alphabet were significantly different. (Duncan’s Multiple Range Test at *p* < 0.05).

**TABLE 2:** The induction of multiple shoots and roots from in vitro shoot explants after 16 weeks of inoculation in MS medium supplemented with BAP.

Concentration of BAP (mg/L)	Percentage of explant regeneration (%)	Average no. of shoots per explant, *N* (*x* ± s.e)	Average no. of roots per explant, *N* (*x* ± s.e)
0.0	21.88	1.57 ± 0.43^d^	4.82 ± 0.3^9^a
0.5	68.75	2.77 ± 0.50^cd^	2.14 ± 0.21^bc^
1.0	75.00	4.29 ± 0.67^bc^	2.56 ± 0.24^b^
1.5	56.25	6.33 ± 0.62^ab^	1.80 ± 0.37^bc^
2.0	84.38	6.85 ± 0.61^a^	0.00 ± 0.00^c^
2.5	87.50	5.11 ± 0.70^abc^	2.00 ± 0.58^bc^
3.0	84.38	6.00 ± 0.85^ab^	0.00 ± 0.00^c^

*Notes:*

* Data followed by different alphabet were significantly different. (Duncan’s Multiple Range Test at *p* < 0.05).

**TABLE 3:** The induction of multiple shoots and roots from in vitro shoot explants after 16 weeks of inoculation in MS medium supplemented with BAP.

Concentration of Kinetin (mg/L)	Percentage of explant regeneration (%)	Average no. of shoots, N (*x* ± s.e)	Average no. of roots, N (*x* ± s.e)
0.0	90.63	2.50 ± 0.22^ab^	4.62 ± 0.35^b^
0.5	75.00	1.36 ± 0.28^b^	6.08 ± 0.54^a^
1.0	78.13	2.70 ± 0.85^ab^	5.29 ± 0.34^ab^
1.5	68.75	1.33 ± 0.33^b^	6.09 ± 0.46^a^
2.0	78.13	2.00 ± 0.71^ab^	4.80 ± 0.44^ab^
2.5	87.50	3.50 ± 0.96^a^	4.96 ± 0.37^ab^
3.0	81.25	1.33 ± 0.33^b^	4.67 ± 0.68^b^

*Notes:*

* Data followed by different alphabet were significantly different. (Duncan’s Multiple Range Test at *p* < 0.05).
